# Screening for lung fibrosis using serum surfactant protein-D, KL-6, and a deep learning algorithm on chest radiographs: a prospective observational study

**DOI:** 10.1186/s12890-025-04062-5

**Published:** 2025-12-17

**Authors:** Hirotaka Nishikiori, Naoya Yama, Kenichi Hirota, Yuki Mori, Ippei Neriai, Haruka Takenaka, Atsushi Saito, Mamoru Takahashi, Koji Kuronuma, Shinichiro Ueda, Masamitsu Hatakenaka, Hirofumi Chiba

**Affiliations:** 1 Department of Respiratory Medicine and Allergology, Sapporo Medical University School of Medicine, South 1-West 16, Chuo-ku, Sapporo, Hokkaido 0608543 Japan; 2 Department of Diagnostic Radiology, Sapporo Medical University School of Medicine, South 1-West 16, Chuo-ku, Sapporo, Hokkaido 0608543 Japan; 3 Scholarly Communication Center Planning and Development Office, Sapporo Medical University, South 1-West 16, Chuo-ku, Sapporo, Hokkaido 0608543 Japan; 4 Department of Clinical Research Education and Management, University of Ryukyus Graduate School of Medicine, 207 Uebaru Nishihara Town, Okinawa, 9030215 Japan

**Keywords:** Lung fibrosis, Interstitial lung disease, Pulmonary fibrosis, Serum biomarker, Artificial intelligence, Deep learning, Health checkup, Interstitial lung abnormality

## Abstract

**Background:**

Early identification of lung fibrosis remains difficult. In Japan, the serum biomarkers surfactant protein-D (SP-D) and KL-6 are commonly used to monitor interstitial lung diseases (ILD) in clinical practice, but their potential role in the early detection of lung fibrosis has not yet been fully clarified. Although chest radiography is also considered a possible tool for identifying subclinical pulmonary fibrosis, detecting early-stage disease remains challenging. A deep learning-based software, BMAX, was recently developed to identify fibrosing ILD on chest radiographs. Its capability to detect lung fibrosis in a health-checkup setting requires validation.

**Methods:**

Study participants were randomly recruited from individuals undergoing routine health examinations. All participants underwent chest radiography and serum SP-D and KL-6 testing. Those with elevated biomarker levels (≥ 110 ng/mL for SP-D and ≥ 500 IU/mL for KL-6) or radiographic abnormalities were advised to undergo further evaluation with chest computed tomography (CT). Lung fibrosis on CT was assessed independently by one pulmonologist and one thoracic radiologist. BMAX assigned a confidence score for lung fibrosis (ranging from 0 to 1) on each radiograph. In participants who underwent CT, the sensitivity and specificity of BMAX (using a confidence score > 0.3 as the threshold), SP-D, and KL-6 for detecting lung fibrosis were evaluated.

**Results:**

Among the 2,751 individuals enrolled, 228 were recommended for CT, and 81 underwent the scan. Lung fibrosis was identified on chest CT in 8 of the 81 participants. The positivity rates for SP-D, KL-6, and BMAX (confidence score > 0.3) were 5.9%, 2.4%, and 5.9%, respectively. SP-D showed a sensitivity of 1.000 and a specificity of 0.315, while KL-6 showed a sensitivity of 0.750 and a specificity of 0.753. BMAX demonstrated a sensitivity of 1.000 and a specificity of 0.904.

**Conclusions:**

SP-D and KL-6 may be useful screening biomarkers for lung fibrosis in health checkup settings, offering high sensitivity and moderate positivity rates. BMAX also appears promising as a standalone screening tool for detecting lung fibrosis on chest radiographs.

## Background

Lung fibrosis often progresses over time in patients with idiopathic pulmonary fibrosis (IPF) and other forms of fibrosing interstitial lung diseases (ILD) [1, 2]. Although antifibrotic drugs can reduce the annual decline in forced vital capacity by approximately 50% in individuals with IPF and other progressive pulmonary fibrosis [1, 3–6], they typically do not completely halt disease progression. In recent years, subclinical lung fibrosis has been increasingly identified through chest computed tomography (CT), with reported progression rates ranging from 20 to 76% [7–9]. These observations underscore the importance of detecting fibrosing ILD at an early stage and initiating antifibrotic therapy at the appropriate time to improve patient outcomes.

Several potential approaches have been proposed for the early detection of lung fibrosis. Fine crackles heard on auscultation are found in over 95% of patients with pulmonary fibrosis, including IPF, and may serve as a useful early indicator [10–12]. In addition, certain circulating biomarkers are known to be elevated in individuals with pulmonary fibrosis. Serum levels of surfactant protein-D (SP-D) and KL-6 have been extensively studied for differentiating ILD patients from healthy individuals and for predicting survival in IPF. These biomarkers are routinely used in clinical practice in Japan to monitor disease activity in ILD patients, with established cutoff values of 110 ng/mL for SP-D and 500 IU/mL for KL-6, respectively [12–14]. However, most prior studies have compared biomarker levels between ILD patients and healthy or disease control groups, and no large-scale investigations have assessed these biomarkers in populations with a lower pretest probability of lung fibrosis, such as individuals undergoing routine health checkups. In this study, we evaluated the ability of SP-D and KL-6 to detect lung fibrosis in a health checkup population who underwent initial screening with serum biomarkers and subsequent evaluation with chest CT.

Another key modality for the early detection of lung fibrosis is medical imaging, particularly chest radiography, as chest radiographs are more commonly used than chest CT scans for screening purposes. However, identifying lung fibrosis at an early stage using chest radiographs remains difficult [15]. To address this challenge, we previously developed a deep learning algorithm designed to detect fibrosing ILD on chest radiographs [16]. Based on our research, a computer-aided detection (CAD) software named BMAX (Registered MAH: Cosmotec, Co., Ltd., Tokyo, Japan) was developed, obtained pharmaceutical approval, and is now commercially available in Japan [15]. This software generates a confidence score ranging from 0 to 1—where 1 indicates the highest level of confidence—reflecting the likelihood that a given radiograph originates from a patient with fibrosing ILD. Although the training data for this algorithm were originally obtained from patients diagnosed with fibrosing ILD, the validation dataset included subclinical participants with CT-detected lung fibrosis which was possible interstitial lung abnormalities (ILA) [16]. However, BMAX as a standalone screening tool for subclinical individuals with early lung fibrosis remains uncertain. This study aimed to validate whether BMAX can effectively detect lung fibrosis among participants in a health checkup who underwent secondary screening with chest CT.

## Methods

### Participants

This study randomly recruited individuals aged 50–100 years who underwent routine health checkups at the Hokkaido Cancer Society (Sapporo, Japan), the Hokkaido Anti-Tuberculosis Association (Sapporo, Japan), and Shin-yurigaoka General Hospital (Kawasaki, Japan) between June 2021 and June 2023. The standard health checkup typically included a basic medical interview that collected information on smoking history, as well as chest radiography, electrocardiography, and urine and blood tests. For the purposes of this study, serum levels of SP-D and KL-6 were also measured.

This study was conducted in accordance with the ethical principles outlined in the Declaration of Helsinki and was approved by the ethics committee of the Sapporo Medical University School of Medicine (approval date, October 28, 2020; approval number, 2–1–42). Written informed consent was obtained from all participants.

The study was registered with the UMIN Clinical Trials Registry (UMIN-CTR; Registration number: UMIN000043855).

### Participants referred for second screening with chest CT scan

**Fig. 1:**
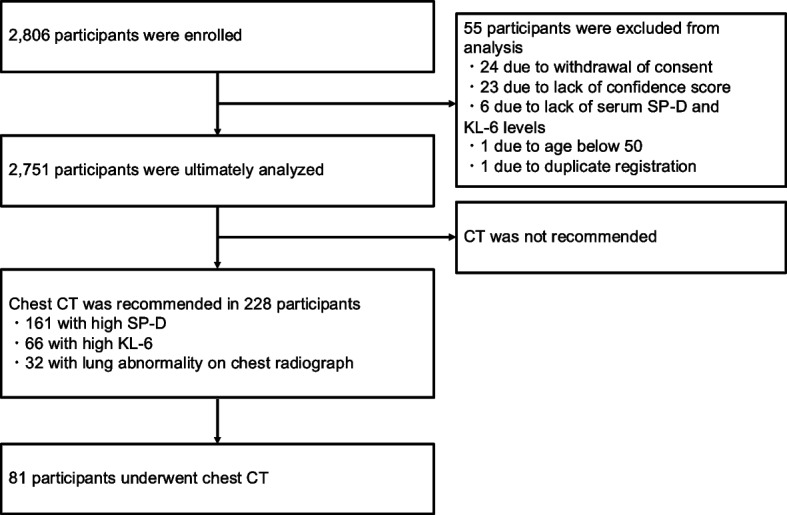
Participant disposition and flow diagram of the study process. CT, computed tomography; SP-D, surfactant protein-D

As part of the standard procedure, chest radiographs obtained during the health checkup were reviewed by routinely assigned radiograph interpreters at each health-check facility to determine whether any abnormalities were present that warranted further evaluation. Participants whose chest radiographs were judged by the interpreters to require additional investigation were advised to undergo a second screening using chest CT. Testing laboratories contracted by each facility measured the serum SP-D and KL-6 levels. Participants with serum SP-D and/or KL-6 levels exceeding the respective cutoff values (110 ng/mL for SP-D and 500 IU/mL for KL-6) were also recommended for second screening with chest CT. The research flow is illustrated in Fig. 1.

### Interpretation of chest CT

Chest CT images obtained during the second screening were sent to the central research institution at Sapporo Medical University, where they were independently interpreted by two ILD specialists— one pulmonologist and one thoracic radiologist, both with more than 20 years of experience. Each specialist assessed the presence of lung fibrosis based on the Fleischner Society position paper on ILA [17] and a representative review on ILA [18]. Although honeycombing and reticulation with traction bronchiectasis and/or lung distortion were interpreted as lung fibrosis, pure ground-glass attenuation without accompanying fibrotic features were not considered indicative of lung fibrosis. The final determination of lung fibrosis was established only when both interpreters independently arrived at the same conclusion.

### Detection capability of serum SP-D and KL-6

Among participants who underwent chest CT, the sensitivity, specificity, and positive predictive values of serum SP-D and KL-6 were calculated based on their respective cutoff values. Additionally, receiver operating characteristic (ROC) analysis was conducted to assess the detection capability using the continuous values of serum SP-D and KL-6, and the area under the ROC curve (AUC) was determined.

### Estimated prevalence of lung fibrosis

To assess whether the prevalence of lung fibrosis inferred through the two serum biomarkers was appropriate, we estimated the prevalence within this cohort using the positivity rates and positive predictive values of serum SP-D and KL-6. The estimated prevalence per 100,000 individuals was calculated using the following formula: (positivity rate of SP-D/KL-6) × (positive predictive value of SP-D/KL-6) × 100,000.

### Detection capability of BMAX

BMAX independently assigned a confidence scores between 0 and 1 to each participant’s chest radiograph, with 1 indicating the highest confidence level for the presence of lung fibrosis [15, 16]. For participants who underwent chest CT, the standalone detection capability of BMAX for lung fibrosis was evaluated. The sensitivity, specificity, and positive predictive values were calculated using a confidence score threshold of 0.3, which was previously applied in studies investigating the detection of fibrosing ILD [15, 16]. ROC analysis was also carried out using the continuous confidence scores.

### Correlations between serum biomarker levels and confidence score

The relationships between serum SP-D/KL-6 levels and the BMAX confidence score were examined across all participants. Since 94% of participants had a confidence score below the threshold of 0.3—suggesting that the majority likely did not have lung fibrosis—correlations were also assessed specifically among those whose confidence scores exceeded 0.3.

### Statistical analysis

Spearman’s rank correlation coefficient was used to evaluate the associations between serum biomarker levels and BMAX confidence scores. To compare confidence scores between chest radiographs paired with CT scans positive for lung fibrosis and those with negative CT findings, Student’s *t*-test was applied. A *p-*value of < 0.05 was considered statistically significant. Statistical analyses were performed using IBM SPSS Statistics for Windows, version 27 (IBM Corp., Armonk, NY, USA).

## Results

### Serum SP-D, KL-6 levels, and chest radiograph abnormalities

A total of 2,806 individuals were enrolled in this study, including 1,600 from Hokkaido Cancer Society, 62 from the Japan Anti-Tuberculosis Association, and 1,144 from Shin-yurigaoka General Hospital. Fifty-five participants were excluded for the following reasons: withdrawal of consent (*n* = 24), missing confidence score data (including absence of chest radiographs; *n* = 23), missing serum SP-D and KL-6 measurements (*n* = 6), age < 50 years (*n* = 1), and duplicate registration (*n* = 1). Consequently, data from 2,751 participants were included in the final analysis (Fig. 1).

Participant characteristics are summarized in Table 1. Serum SP-D and KL-6 levels exceeded their respective cutoff values in 161 participants (5.9%) and 66 participants (2.4%). Abnormalities on chest radiographs deemed to require a second screening were identified by the attending interpreters in 32 participants.

**Table 1:** Participant characteristics, serum biomarkers, and confidence score

	**Entire cohort**	**CT cohort**^**#**^	**Fibrosis cohort**^**$**^
Number of participants	2,751	81	8
Age (years)	58.9 (6.6)	63.0 (8.7)	68.6 (4.4)
Sex, female/male	1,163/1,588	31/50	1/7
BMI	23.60 (3.75)	23.44 (3.69)	23.78 (3.10)
Smoking history never/ex/current/unknown	1,008/925/779/39	26/25/28/2	1/4/3/0
Serum SP-D level (ng/mL)	50.2 (33.5)	126.0 (60.2)	195.3 (93.2)
Serum SP-D level ≥ 110 ng/mL (Y/N)	161/2,590	58/23	8/0
Serum KL-6 level (U/mL)	238.4 (112.1)	419.7 (363.3)	868.9 (779.2)
Serum KL-6 level ≥ 500 U/mL (Y/N)	66/2,685	24/57	6/2
BMAX confidence score	0.083 (0.119)	0.188 (0.247)	0.720 (0.233)
Confidence score > 0.3 (Y/N)	161/2,590	15/66	8/0
Abnormality on chest radiograph* (Y/N)	32/2,719	8/73	0/8

Values are presented as mean (standard deviation) or number of participants

*BMI* body mass index, *CT* computed tomography, *SP-D* surfactant protein-D, *Y* yes, *N* no

^#^Indicates the subset of participants who underwent chest CT

^$^Indicates the subset with lung fibrosis confirmed by chest CT

^*^Refers to abnormalities on chest radiographs judged to require second screening by the attending interpreters at each health checkup facility

### Signs of lung fibrosis on chest CT

A total of 228 participants were advised to undergo second screening with chest CT, and 81 ultimately completed the scan. The characteristics of these participants are summarized in Table 1. Among the 81 chest CT scans, 11 were performed with section thickness ≤ 1.5 mm and 75 with section thickness ≤ 5 mm. Of those scanned, 58 had elevated SP-D levels, 24 had elevated KL-6 levels, and 8 exhibited chest radiograph abnormalities that prompted the recommendation for further screening.

**Fig. 2:**
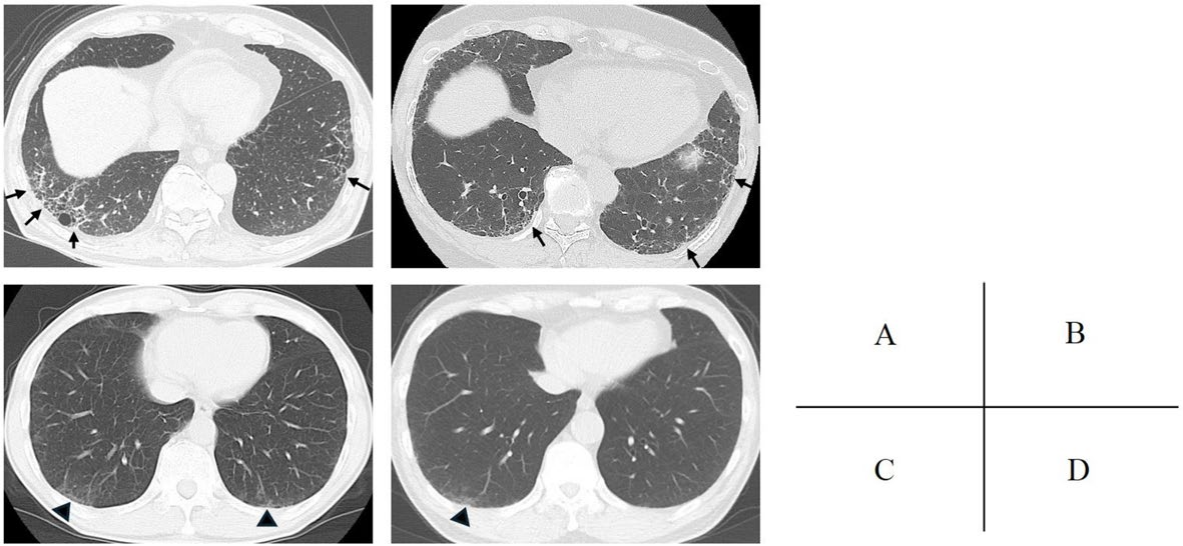
Example chest CT images. Panels **a** and **b** shows images from a 75-year-old male (current smoker) and a 64-year-old female (ex-smoker), respectively, both assessed as positive for lung fibrosis by both interpreters. Subpleural reticular opacities are visible in the lower lobes bilaterally (arrows). Panels **c** and **d** display images from a 72-year-old male (current smoker) and a 63-year-old male (ex-smoker), respectively, assessed as negative for lung fibrosis. Subtle subpleural pure ground-glass opacities are present (arrowheads). CT, computed tomography

Among the individuals who underwent chest CT, signs of lung fibrosis were identified by both interpreters in eight cases. The concordance rate between the two interpreters in diagnosing lung fibrosis was 82.7%. The characteristics of the participants with CT-confirmed lung fibrosis are also shown in Table 1, and representative CT images are provided in Fig. 2. Of the eight individuals identified as having lung fibrosis, only one was female, and only one had a prior diagnosis of ILD; this individual was also the only never-smoker among the group.

### Detection capability of serum SP-D and KL-6

**Fig. 3:**
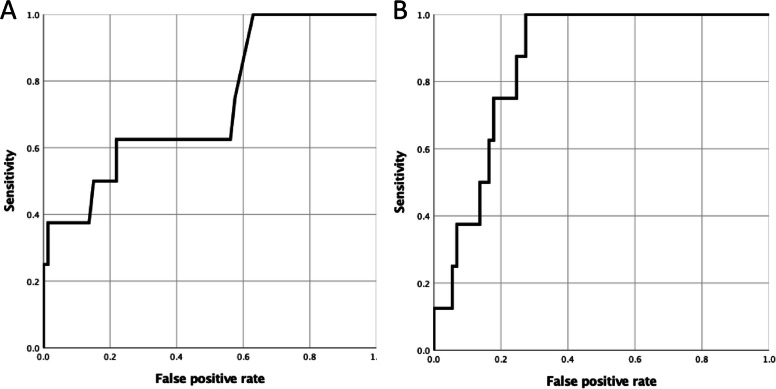
ROC curves showing the performance of serum surfactant protein-D (**a**) and KL-6 (**b**) in detecting lung fibrosis. ROC, receiver operating characteristic

All participants who showed signs of fibrosis had elevated serum SP-D levels. Among those who underwent chest CT, the sensitivity, specificity, and positive predictive values of serum SP-D for identifying fibrosis were 1.000 (95% confidence interval [CI], 0.518–1.000), 0.315 (0.211–0.434), and 0.138 (0.061–0.254), respectively. Similarly, elevated serum KL-6 levels were observed in six participants, with a sensitivity of 0.750 (0.349–0.968), specificity of 0.753 (0.639–0.847), and positive predictive value of 0.250 (0.098–0.467). The AUC for serum SP-D and KL-6 levels was 0.731 (0.542–0.921; Fig. 3a) and 0.860 (0.771–0.948; Fig. 3b), respectively. None of the participants who had chest radiograph abnormalities prompting second screening were found to have fibrosis on their chest CT scans.

### Estimated prevalence of lung fibrosis

Using the positivity rate and positive predictive value of SP-D, the estimated prevalence of lung fibrosis was calculated to be 807.2 per 100,000 individuals. Based on the KL-6 positivity rate and positive predictive value, the estimated prevalence was 599.8 per 100,000 individuals.

### Detection capability of BMAX

**Fig. 4:**
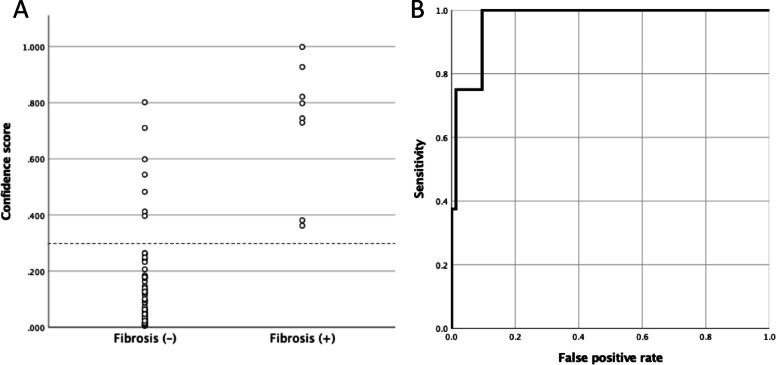
Confidence score distribution among participants who underwent chest CT (**a**) and ROC curve illustrating the performance of the BMAX confidence score in detecting lung fibrosis (**b**). CT, computed tomography; ROC, receiver operating characteristic

A confidence score above 0.3 was recorded in 161 participants (5.9%) across the entire cohort. Among these individuals, 25 had elevated serum SP-D levels, 20 had elevated KL-6 levels, and 12 had elevations in both biomarkers. Of the participants with a confidence score > 0.3, 15 underwent chest CT, and 8 of them showed CT findings consistent with lung fibrosis. Conversely, all individuals exhibiting lung fibrosis on chest CT had confidence scores > 0.3 on their chest radiographs. The distribution of confidence scores is illustrated in Fig. 4a. The confidence scores of radiographs corresponding to CT-positive cases for fibrosis were significantly higher (*p* < 0.001) than those of CT-negative cases among participants who underwent chest CT. The sensitivity, specificity, and positive predictive value of BMAX for detecting signs of fibrosis were 1.000 (95% CI, 0.518–1.000), 0.904 (0.812–0.961), and 0.533 (0.266–0.787), respectively. Based on the continuous confidence score, the AUC was 0.971 (0.934–1.000) (Fig. 4b).

### Correlations between serum biomarker levels and confidence score

**Fig. 5:**
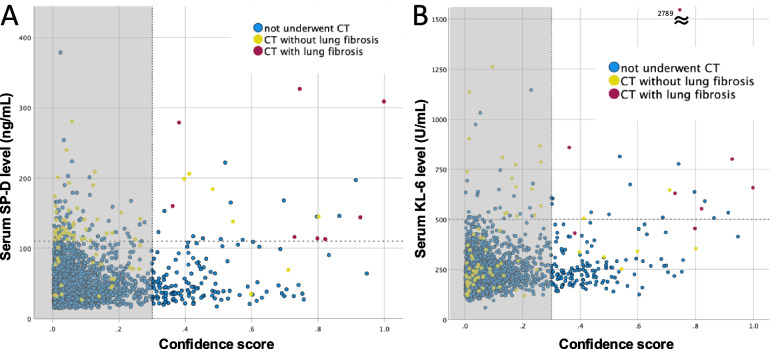
Correlation between serum SP-D (**a**) and KL-6 (**b**) levels and the BMAX confidence score. CT, computed tomography; SP-D, surfactant protein-D. Patients with confidence score above 0.3 are plotted outside the gray bundle. Red plots indicate participants with CT-confirmed lung fibrosis; yellow plots represent those without fibrosis; blue plots indicate participants who did not undergo chest CT

The correlation coefficients between the BMAX confidence score and serum levels of SP-D and KL-6 were 0.086 (*p* < 0.001) and 0.204 (*p* < 0.001), respectively (Fig. 5). Among participants with a confidence score exceeding the 0.3 threshold, serum SP-D and KL-6 levels showed modest correlations with the confidence score, with correlation coefficients of 0.313 (*p* < 0.001) and 0.338 (*p* < 0.001), respectively (as shown outside the gray band in Fig. 5).

## Discussion

In this study, out of 2,751 individuals who underwent routine health checkups, 81 participants—screened based on serum SP-D and KL-6 levels along with routine interpretation of chest radiographs—proceeded to chest CT as a second screening step. Among these 81 CT scans, 8 were confirmed to show signs of lung fibrosis. In this subset, the sensitivities of serum SP-D and KL-6 for detecting lung fibrosis were 1.000 and 0.750, respectively. The AUC for serum SP-D and KL-6 were 0.731 and 0.860, respectively. Across the entire cohort, the positive rates for SP-D and KL-6 were 5.9% and 2.4%, respectively. Considering their high sensitivities and reasonable positivity rates, both serum biomarkers appear suitable for use in lung fibrosis screening during health checkups.

Takahashi et al. reported that a serum SP-D cutoff of 110 ng/mL could detect IPF with a sensitivity of 84.6% and a specificity of 95.4% [13]. Ohnishi et al. found that a KL-6 cutoff value of 465 U/mL could detect ILDs with 93.9% sensitivity and 96.3% specificity [14]. In a large cohort of IPF patients registered in Japan’s medical subsidy system for intractable diseases, 89.3% and 92.6% of all patients—and even 84.7% and 85.9% of those with the mildest disease (Stage I of the Japanese severity classification [19])—had serum SP-D and KL-6 levels exceeding the respective cutoff values of 110 ng/mL and 500 IU/mL [12]. These findings suggest that both serum biomarkers may not only detect IPF with high sensitivity but also contribute to early detection. Furthermore, a study by Kashiwabara demonstrated that serum levels of KL-6, SP-D, and surfactant protein-A were significantly higher in individuals with fine parenchymal abnormalities confirmed on prone CT compared to those whose abnormalities resolved in the prone position [20], indicating that these biomarkers may be capable of identifying subtle fibrotic changes in the lung.

As the use of CT imaging becomes more widespread, subclinical abnormalities are increasingly being identified in clinical practice. Interstitial abnormalities detected in individuals without respiratory symptoms or measurable lung function impairment are referred to as ILA [17]. Individuals with ILA have been shown to exhibit reduced diffusing capacity for carbon monoxide and lower total lung capacity compared to those without ILA [21]. Moreover, approximately 76% of individuals with ILA demonstrated progression over a period of about 6 years, and this group had a worse prognosis than those whose ILA did not progress [7]. Although ILA may include findings such as ground-glass attenuation without fibrotic features, the presence of fibrotic changes—such as subpleural reticulation accompanied by lung distortion and traction bronchiectasis—has a stronger association with future disease progression than pure ground-glass attenuation alone [8]. Therefore, identifying fibrotic features is critical for recognizing individuals at risk of developing overt fibrosing ILD. The reported prevalence of ILA varies depending on the population studied and the duration of observation. Jin et al. reported a 9.7% prevalence of ILA among cigarette smokers, with fibrotic patterns observed in 39.5% of those with ILA—equating to 3.8% of all participants [9]. They also noted progression in 37% of individuals with fibrotic ILA over a 2-year follow-up period. Similarly, Lee et al. assessed the prevalence and types of ILA among individuals aged ≥ 50 years across three health screening centers. ILA was identified in 94 out of 2,765 participants (3%), with signs of fibrosis present in 59 individuals (1.9% of all participants) [22]. In the present study, the estimated prevalence of lung fibrosis was calculated based on the positivity rates and positive predictive values of serum SP-D and KL-6, yielding estimates of 807.2 and 599.8 per 100,000 individuals, respectively. These values were somewhat lower than those reported in previous studies. One possible explanation is that some individuals with lung fibrosis may not have shown elevated serum biomarker levels. Another contributing factor may be the relatively younger age of the study population, which had a mean age of 58.9 years, given that pulmonary fibrosis is more frequently observed in older individuals. A higher prevalence might have been observed if a similar study were conducted in an older cohort.

The detection capability of BMAX, a CAD software developed for identifying fibrosing ILD on chest radiographs, was also evaluated among participants who underwent chest CT. All eight individuals with CT-confirmed signs of lung fibrosis had BMAX confidence scores exceeding 0.3, whereas only 7 out of 73 participants without fibrosis had scores above this threshold. The sensitivity and specificity of BMAX for detecting lung fibrosis were 1.000 and 0.904, respectively. The AUC based on the BMAX score was 0.971. Although this result is limited by the fact that most participants who underwent CT were selected based on elevated serum SP-D and/or KL-6 levels, BMAX may be promising as a standalone screening tool for detecting lung fibrosis on chest radiographs.

Among participants with a confidence score above 0.3, mild correlations were observed between the BMAX confidence score and serum levels of SP-D/KL-6. These findings suggest that the BMAX confidence score, along with serum SP-D and KL-6 levels, may reflect the degree of lung fibrosis. Our previous research also demonstrated a significant association between the BMAX confidence score and the extent of fibrotic changes observed on chest radiographs [15]. Additionally, another study reported a significant association between serum SP-D levels and the extent of disease on chest CT in patients with IPF [13]. These findings support our current hypothesis.

This study has several limitations. First, the relatively small CT subset and the limited number of CT-identified lung fibrosis cases may restrict the generalizability of the findings. Thus, future studies should have a larger proportion of CT-evaluated participants to validate the effectiveness of this screening strategy. Second, not all individuals with elevated serum biomarker levels underwent chest CT, which may have introduced selection bias. Third, whether early lung fibrosis detected by this screening can serve as a predictor of progression to clinically significant ILD remains uncertain. Hence, a longitudinal study is warranted to confirm this. Fourth, since the detection performance of BMAX was evaluated primarily in participants with elevated biomarker levels, it remains uncertain whether BMAX would demonstrate similar accuracy in individuals without such elevations. Moreover, serum SP-D and KL-6 levels are generally elevated in fibrosing ILD; however, the positivity rate and degree of elevation vary depending on ILD subtypes [23–26], and screening using these two biomarkers might not have identified all lung fibrosis cases. Fifth, although most patients with a known ILD diagnosis may not routinely undergo chest radiography as part of health checkups, there were no restrictions preventing such individuals from participating. Therefore, a few patients who had a prior diagnosis of ILD—such as the one case described in the results—might have been included in this study. Finally, most chest CT scans performed during the second screening did not use sub-1.5 mm slice thickness, which is the recommended standard for evaluating ILA [17]. This may have influenced the accuracy of lung fibrosis assessment.

## Conclusions

Serum SP-6 and KL-6 may serve as suitable screening tools for detecting lung fibrosis during health checkups due to their high sensitivities and reasonable positivity rates. BMAX also shows promise as a standalone screening method for identifying lung fibrosis on chest radiographs.

## Data Availability

All datasets are available from the corresponding author upon reasonable request.

## Abbreviations

AUC: Area under the curve
CAD: Computer-aided detection
CI: Confidence interval
CT: Computed tomography
ILA: Interstitial lung abnormalities
ILD: Interstitial lung diseases
IPF: Idiopathic pulmonary fibrosis
ROC: Receiver operating characteristic
SP-D: Surfactant protein-D
